# 

**Published:** 1895-07

**Authors:** 


### Communicated.

### AMERICAN DENTAL ASSOCIATION MEETING.

The Local Committee have made arrangements with the following Hotels and houses for the sessions of the American Dental Association commencing Tuesday, Aug. 6th, 1895. The "Coleman House" fronting the ocean, the largest Hotel in Asbury Park with rates $3.50 and $4.00 per day and the use of the ball room at stated periods of the day and evenings for large Committee meetings and sections. The "West End Hotel" a first-class Hotel opposite the "Coleman House," the rates $2.50 and $3.00 per day with the use of the ball room at stated periods for Committees and sections. The "Ocean Hotel" next door to the "West End" capacity 900, first class in every respect rates based on 100 or more Dental Convention guests at $2.50 and $3.50 per day with the use at stated periods of small parlors for Committees. The "Hotel Brunswick" near the Ocean, first class in every respect from $3.00 per day up to $20. per week. This Hotel has a series of small parlors on the main floor for Committees and private receptions, the use of the same is based on the number of Dental Convention guests at the Hotel.

In the large Hotels the use of the rooms for Committees and sections is based on the contract of a certain number of guests, therefore the selection of a room ahead in some Hotel is necessary, July 15th is the time most of the Hotels would like to know.

All Hotels have electric lights, baths and artesian well water.

In the small hotels the "Grand Central" rates $1.50 to $2.50 per day and $14 to $16 per week. The "Ashland" $2 per day and $8 to $12 per week. The "Portland" $2 per day and $8 to $10 per week. The "Edgemore" $2 to

$2.50 per day and $12 to $20 per week. The "Neptune" a nice quiet place $1.75 to $2 per day and $9 to $20 per week. The "Albany" $2 per day and $8 to $15 per week. The "Clifton" $2 per day and $15 per week. The "Strand" $2 per day and $10 per week.

The Committee will have an attendant at the Auditorium from July 27th to August 9th, to give information in reference to Hotels and other information to enquiring members. The trolley cars run direct from the depot to the Auditorium. A map of Asbury Park the cuts of the Hotels and the Auditorium will appear in the New Jersey Programme which will be mailed to every member of the American Dental Association.

CHAS. A. MEEKER,
C. W. F. HOLBROOK,
C. S. STOCTON,
Chairmen Local Committee.

---

### ODONTOLOGICAL SOCIETY OF CHICAGO.

Resolutions adopted by the Odontological Society of Chicago, in reference to the death of Dr. J. J. R. Patrick, of Belleville, Illinois.

WHEREAS, death has removed from our midst, Dr. John J. B. Patrick, of Bellville, Ill., and there has passed from the scenes of human activity a character whose labors have enriched the stores of science, a man who delved fearlessly and steadfastly into those mysteries of nature which call forth the most subtle energies of the human mind, and yield results making the lives of succeeding generations happier, now therefore be it

RESOLVED, that in the death of Dr. Patrick, the world loses an honored citizen soldier, the field of science a conscientious, faithful laborer, and the dental profession a light whose extinguishment will kindle the profession's interest in the work he has accomplished for it, and thus extend and perpetuate his good influence. Be it further

RESOLVED, that to the family, friends and professional associates of Dr. Patrick, we extend the assurance that we appreciate his worth and are grateful that his life had been spared until it nearly completed the allotted time of three score and ten.

JAMES A. SWASEY,
ALISON W. HARLAN,
LOUIS OTTOFY,
Committee.

---

### AMERICAN DENTAL ASSOCIATION.

The 35th annual meeting of the American Dental Association will be held at Asbury Park, N. J., commencing Tuesday, August 6, 1895, and continuing for four days.

A rate of *fare and one third for the round trip* upon the "certificate plan," has been secured. In order to get this reduction, full fare must be paid in going to the meeting, a *receipt* being obtained therefor from the ticket agent, at the *starting point.* If traveling over more than one line, secure a certificate over each line, or have the ticket agent at the starting point name in the receipt the different roads over which the ticket is good. This receipt (certificate), must be countersigned by the Secretary of the Association, and entitles the holder to return for one-third fare. Arrangements have been made to have the joint agent of the rail-roads present at Asbury Park on Wednesday, August 7, and it is desirable, that all who intend to attend the meeting, shall be in attendance, so that their rail-road certificates can be passed upon at that time. No rates have been granted over the lines comprised in the Western Passenger Association. Members residing west of Chicago should secure the best rates they can to the point, where they enter the territory in which the reduced rate prevails.

J. N. CROUSE,
Chairman of the Executive Committee,
2231 Prairie Avenue, Chicago.

### To Readers and Correspondents.

Communications intended for publication, and exchange journals and papers, should be addressed to the Editor of The American Journal of Dental Science, No. 845 N. Eutaw St. Baltimore, Md. All letters relating to the business of the Journal, or containing cash remittances, to be directed to Snowden & Cowman Mfg. Co., 9 W. Fayette Street, Baltimore, Md. Articles for publication should be received by the Editor at least two weeks previous to the first of the month on which the number in which it is designed for them to appear, is issued.

☞ The American Journal of Dental Science will contain fifty pages of reading matter in each number, making a volume of about Six Hundred pages,

EXCLUSIVE OF ADVERTISEMENTS

